# Parent Support and Parent-Mediated Behaviors Are Associated with Children's Sugary Beverage Consumption

**DOI:** 10.1016/j.jand.2011.11.013

**Published:** 2012-04

**Authors:** Nanette V. Lopez, Guadalupe X. Ayala, Kirsten Corder, Christina M. Eisenberg, Michelle M. Zive, Christine Wood, John P. Elder

**Keywords:** Sugary beverages, Parenting model, Parent support, Parent-mediated behaviors

## Abstract

Consumption of sugary beverages has been identified as a contributor to childhood obesity. Studies have established the importance of specific parenting practices to children's beverage consumption; however, no study has examined multiple operationalizations of parenting to better understand where to focus future interventions. The present study examined the relationship between children's sugary beverage consumption and a parenting model that included household food rules, parent modeling of food rules, parent-mediated behaviors, and parent support. Baseline data from *Project MOVE/me Muevo* were used. Participants included 541 children, aged 5 to 8 years old, and their parents. Parents completed a 45-minute self-administered survey in Spanish or English, providing information about their child's dietary intake, as well as their parenting practices. Children's sugary beverage consumption included nondiet soda, noncarbonated sugary drinks, and sport drinks. Household food rules and parent modeling of food rules were assessed with seven items each. Parent-mediated behaviors consisted of four behaviors. Parent support was assessed with five items. Parent support and parent-mediated behaviors, including total screen time and eating at fast-food restaurants at least weekly, were associated with greater consumption of sugary beverages in children. No other parenting variables were significant. Encouraging caregivers to promote healthy dietary behaviors and provide healthy choices, limiting children's television and computer use, and reducing fast-food consumption can contribute to reductions in sugary beverage consumption among children.

Consumption of sugary beverages is a contributor to childhood obesity (1-4). The Academy of Nutrition and Dietetics recommends that total added sugars not exceed 25% of a child's total daily caloric intake (5). In addition, the American Academy of Pediatrics recommends limiting fruit juice consumption to 4 to 6 oz/day for children ages 1 through 6 years and 8 to 12 oz/day for older children (6).

Within an ecological framework, parents play a role in children's behaviors. In particular, parents are ultimately responsible for their children's food and beverage choices because young children have little control over these purchases. Given their importance, researchers have examined ways in which parents can influence beverage consumption (7-9). Specific parenting practices include rule setting (10), parent modeling of or adherence to rules (11), parent-mediated behaviors (12), and parent support (13).

Increasing consumption of sugary beverages warrants examination of correlates to prevent additional increases. Numerous studies have identified the importance of specific parenting practices to children's sugary beverage consumption. This study extends this research by testing a parenting model for children's sugary beverage consumption. In this study, sugary beverages included nondiet soda, noncarbonated sugary drinks, and sport drinks. Davison and Campbell identified four categories of parenting related to children's obesity risk behaviors: beliefs and knowledge, modeling, accessibility, and shaping (14). We specifically examined the relationship between children's sugary beverage consumption and these four parenting categories: household food rules, parent modeling of food rules, parent-mediated behaviors, and parent support for healthy eating.

## Methods

This cross-sectional study used baseline data from *Project MOVE/me Muevo*, a recreation center-based obesity-prevention intervention for children. Institutional Review Board approval was obtained from San Diego State University. Participants included 541 children, aged 5 to 8 years old, and their parents living in San Diego County, CA. Parents were required to be the participating child's legal guardian or primary caregiver. Between November 2006 and May 2008, families were recruited through targeted phone calls and at public locations, community events, and the 30 participating recreation centers. One parent/legal guardian provided written informed consent, with the child providing verbal assent.

### Procedures

Parents completed a 45-minute self-administered survey in Spanish or English. All measures were conducted between April 2007 and May 2008.

#### Children's Sugary Beverage Intake

Children's sugary beverage intake was assessed using a previously validated scale (15) and included nondiet soda, noncarbonated sugary drinks, and sport drinks. Response options consisted of common beverage portions and cup sizes. For example, soda consumption was assessed according to frequency of consumption using a 12-oz. can/glass as never/less than 1 per month, 1 to 3 cans/glasses per month, 1 can/glass per week, 2 to 6 cans/glasses per week, 1 can/glass per day, or 2 or more cans/glasses per day. Item responses were converted to mean daily servings and then summed, with higher scores representing greater daily consumption of sugary beverages.

#### Household Food Rules

Household food rules regarding diet were assessed using five items from Active Where (16), plus two developed by the study team using data from *Aventuras para Niños* (17) (Table 1). “Sometimes” responses were recoded into “yes” responses because any enforcement of rules could affect a child's diet. Table 1 lists the test-retest reliability data for the five household rules used from Active Where (18). A final score was computed by summing affirmative responses with a higher score indicating more household food rules.

#### Parent Modeling of Food Rules

Parents were assessed on whether they followed the same seven household food rules set for their children, modified to reflect parent behavior. The same response options and recoding were used.

#### Parent-Mediated Behaviors

Four parent-mediated behaviors were examined: the frequency of family dinner eaten together, frequency of eating away-from-home meals, frequency of the child eating or snacking while watching television, and total amount of screen time per day.

Frequency of family dinner eaten together was assessed using one item from a previous study conducted with the target population (19) (Table 1). Responses were recoded into mean times per week. For example, “5 to 7 times a week” was recoded into 6 times a week. “Less than once a week” and “1 to 2 times a week” were collapsed into one response in order to approximate equal distribution between response categories. This grouping resulted in the creation of three response categories: 2 or less times per week, 3.5 times per week, and 6 times per week.

Frequency of eating away-from-home meals was assessed using three items from a previous study targeting the same population (20) (Table 1). For each item, five response options were provided and recoded as “never/less than once a week” or “once a week or more,” based on evidence that at least weekly consumption of prepared foods purchased outside the home is associated with poorer diet quality (20).

Weekly frequency of the child eating or snacking while watching television was assessed using three items from a previous study targeting the same population (21) (Table 1). Responses were recoded into mean times per week. Responses to all three questions were used to create a summary score, such that a higher score indicated a greater number of days per week engaged in these behaviors.

Total daily screen time was assessed using three items used in the Active Where study (16) (Table 1). A total sum score of daily screen time was computed with higher scores reflecting more minutes of screen time. Table 1 lists the test-retest reliability for the screen time variables used in Active Where (18).

#### Parent Support

Social support was assessed with five items used in the Patient-centered Assessment and Counseling for Exercise plus Nutrition (PACE+) study (22) (Table 1). Response options and recoding were identical to those for weekly frequency of the child eating or snacking while watching television. Responses were collapsed into one summary score, such that a higher score indicated a greater number of days of parent support in a typical week.

### Demographics

Parent/primary caregiver and child demographics included age, sex, and ethnicity, with parents/primary caregivers reporting monthly family income before taxes from all sources and highest level of education completed. Parent/primary caregiver's and child's ethnicity was assessed by asking whether or not he/she considered himself/herself and his/her child Latino, Hispanic, Mexican/Mexican American, or of Spanish origin. Total monthly family income before taxes was recoded into $0 to $2,000; $2,001 to $3,500; $3,501 to $5,000; and $5,001 or more. Caregiver education level was categorized as middle school or less, high school, some college, college graduate, and postgraduate work.

### Statistical Analysis

Analyses were conducted using PASW Statistics 18.0 (2009, SPSS Inc, Chicago, IL). Descriptive statistics included means and standard deviations for continuous data and frequencies for categorical data. Bivariate analyses examined correlations between individual scale items and children's sugary beverage consumption, with no variations found in the direction of associations among items within the same construct. Therefore, a multiple linear regression analysis determined the relative contribution of household food rules, parent modeling of food rules, parent-mediated behaviors, and parent support to children's sugary beverage consumption. The dependent variable was not normally distributed so the variable was log plus one transformed. The regression analysis included five blocks of variable groupings in accordance with the proposed parent model. The first block consisted of demographics such as caregiver's age and education, and child sex. The second block included Household Food Rules, the third block included Parent Modeling of Food Rules, the fourth block included Parent-Mediated Behaviors, and the final block included Parent Support. The fifth block was used to interpret the independent associations of the variable groups and of the total model. Blocks were ordered based on the relative contribution to children's beverage consumption. For example, household food rules, followed by parent modeling of food rules, and parent support were found to have a decreasing effect on the body mass index (calculated as kg/m^2^) of girls in a 5-year longitudinal study (23).

## Results and Discussion

Caregiver demographics indicated 93% were female, 41% were Latino/Hispanic, and mean age was 37.6 (±6.5) years. Among the caregivers, 30.1% completed high school or less and 43.1% completed college or postgraduate work. Child demographics indicated 55.1% were female, 46.0% were Latino/Hispanic, and mean age was 6.7 (±0.7) years. Descriptive statistics indicated children consumed a mean of 0.51 (±0.58) daily servings of sugary beverages and spent 108.0 (±86.2) minutes in total daily screen time. Parent household food rules, modeling of household food rules, and parent support sum scores were 4.7±1.8, 5.2±1.7, and 5.2±1.5, respectively. Results from the hierarchical regression analysis examining correlates of the log-transformed sugary beverage consumption are in Table 2. Due to missing data on some of the parenting variables included in the regression analysis, the final analytic sample was 539. In the demographics block, significant negative associations were found between sugary beverage consumption and caregiver education (*P*≤0.01), such that with more education, caregivers reported less consumption of sugary beverages in their children. In Block 2, having more household food rules was negatively associated with sugary beverage consumption; however, this association was no longer significant in the full model. In Block 4, a significant positive association was found between sugary beverage consumption and weekly visits to fast-food restaurants (*P*≤0.05) and total screen time (*P*≤0.05). In Block 5, a significant negative association was found between sugary beverage consumption and parent support (*P*≤0.001). The positive associations between sugary beverage consumption and weekly visits to fast-food restaurants and total screen time remained in Block 5. Greater parent support was associated with less consumption of sugary beverages. Eating away-from-home meals at fast-food restaurants at least weekly and more screen time were both associated with greater consumption of sugary beverages. No other relationships were significant.

This study tested the relationship between a parenting model that included household food rules, parent modeling of food rules, parent-mediated behaviors, and parent support with children's sugary beverage consumption. Children consumed, on average, half a serving of sugary beverages per day, less than an elementary school sample reporting an average of one daily serving (24). Consumption in the present study might be lower because of the younger age of the children compared with the elementary school sample. As children age, sugary beverage consumption increases (25). Total mean daily screen time was 108.0 minutes, with 30.3% of the caregivers reporting their child spent 2 hours or more in front of a screen. These children accumulated less daily screen time compared with national data that indicated nearly 50% of girls and 55% of boys aged 6 to 11 years old spend ≥2 hours in front of a screen every day (26). This could be a result of the larger age range in the national sample because children engage in more screen time as they get older (27).

Regression analyses indicated that parent-mediated behaviors were associated with greater consumption of children's sugary beverages. These results match previous studies that showed television viewing was associated with consumption of high-energy drinks among 6-year-old Australians (28). In a cross-sectional study with school-aged children living in Maryland, results indicated that those who lived in high television-viewing families consumed 5% more of their total daily energy intake from soda (29). This might be a result of the effects of television advertising (30). Data from a 2008 study indicated that all 27 beverage advertisements shown during Saturday morning children's programming promoted choices that do not meet nutrition standards (31).

In addition to total screen time, eating away-from-home meals at fast-food restaurants was positively associated with children's sugary beverage consumption. This supports previous findings in which eating away-from-home meals at least once a week or more was associated with greater consumption of sugary beverages (20). Additional research indicates that visits to fast-food restaurants are positively associated with sugary beverage consumption (32,33).

Parent support was negatively associated with consumption of sugary beverages; in other words, greater parent support for healthy eating was associated with less sugary beverage consumption. Home availability is one aspect of parental social support. One study found a positive relationship between availability of soft drinks in the home and consumption of soft drinks in a sample of 8- to 13-year-old children (11). Unfortunately, additional research examining social support between parents and elementary school-aged children is unavailable.

Results from the current study were inconsistent with previous studies. For example, previous research shows that eating dinner together as a family (34) and less frequent television watching when eating meals (35) are associated with less consumption of sugary beverages among children. These associations were not observed in the present study and could be explained by the fact that the current study involved younger children compared with those in previous studies. The current study does support the lack of associations found between other parenting constructs, such as household rules and parent modeling of rules and sugary beverage consumption among children (28).

Current study limitations include a cross-sectional study design, a finite number of categories for beverage serving sizes, which prevents detailed measurement, limitations in what parents/caregivers know about what children are consuming throughout the day, and potential self-report bias, resulting from recall issues and social desirability. Longitudinal studies are needed to determine whether the constructs are individually or collectively predictive of children's beverage consumption. Parent report serves as a proxy for child beverage consumption, screen time, and family meal behaviors because of the children's young age, with parents possibly having difficulty recalling consumption of all beverages because of consumption of beverages when away from the parent or inability to accurately report quantities (36). More precise measures of overall diet include direct observation, doubly labeled water, 24-hour recall, and food frequency questionnaires. The only method used to assess diet in the current study was a survey.

Study strengths include a large sample size (n=541) and an ethnically diverse sample (41% Latino) consistent with San Diego County census data, which indicated that 31% of residents are of Hispanic/Latino origin (37). In addition, in terms of primary caregiver education, 24.6% reported graduating from college compared with 34.0% of county residents who are college graduates. Although median income in San Diego County is $60,103, this sample reflects an overall lower median income of $42,000 to $48,000 as reported by primary caregivers. This lower income level might be a result of the lower percentage of college graduates in the current study. Research indicates that mothers with less education have higher emotional feeding scores compared with mothers with more education (38), meaning they provide food as a form of comfort in the absence of hunger. This might contribute to consumption of unhealthy foods and beverages in children with less-educated mothers.

## Conclusions

Current study results can inform future interventions by highlighting correlates of sugary beverage consumption, which is related to childhood obesity. Parent behaviors, including limiting screen time and eating away-from-home meals at fast-food restaurants, were associated with sugary beverage consumption and can be promising avenues for obesity prevention. The American Academy of Pediatrics recommends limiting screen time to ≤2 hours per day for children 2 years of age and older (39). Public health advocates can use these guidelines to inform parents about their children's screen time behaviors. Parent support, including reducing the availability and accessibility of sugary beverages, could also limit opportunities for sugary beverage consumption.

## Figures and Tables

**Table 1:** Parenting constructs and individual items used on Project MOVE/me Muevo baseline survey (n=541)

Construct	Item	Response options	ICC^a^ (18)
Household rules Cronbach's α=.68	Limited portion sizes at meals (16)	Yes, no, sometimes	.608
	No meals with the TV^b^/DVD^c^ on (16)		.694
	No fried snacks (such as potato chips) at home (16)		.736
	Must eat dinner with family (16)		.618
	Limited fast food (16)		.703
	No sugary beverages (17)		NA^d^
	Must finish all food on plate (17)		NA
Parent modeling of rules Cronbach's α=.66	Same items as above, modified to reflect parent behavior	Yes, no, sometimes	NA
Parent-mediated behaviors	Frequency of family dinner eaten together (19) In a typical week, how often does your family eat dinner together?	Less than once a week, 1 to 2 times a week, 3 to 4 times a week, 5 to 7 times a week	NA
Cronbach's α=.68	Frequency of eating away-from-home meals (20) How often does the family usually go out to eat or bring home ready-to-eat foods from …?: 1) Relatives' or friends' homes 2) Fast-food restaurants 3) Other restaurants including sit-down restaurants	Never, less than once a week, 1 to 2 times per week, 3 to 4 times per week, 5 or more times per week	NA
Cronbach's α=.70	Frequency of child eating or snacking while watching TV (21) 1) How often is the TV on when the family is eating dinner? 2) How often does your child eat snacks in front of the TV? 3) How often does your child eat meals in front of the TV?	Never, 1 to 2 days, 3 to 4 days, 5 to 6 days, Everyday	NA
Total amount of daily screen time Cronbach's α=.49	On a typical weekday, how much time does your child spend …? (16): 1) Watching television/videos/DVDs 2) Playing computer or video games (eg, Nintendo [Nintendo Co, Ltd, Kyoto, Japan] or Xbox [Microsoft Corp, Redmond, WA]) 3) Using the Internet, e-mail, or other electronic media for leisure	None, 15 min, 30 min, 1 hour, 2 hours, 3 hours, 4 hours or more	1) .665 2) .729 3) .715
Parent support Cronbach's α=.68	During a typical week, on how many days does an adult member of your household …? (22):	Never, 1 to 2 days, 3 to 4 days, 5 to 6 days, everyday	NA
	Encourage your child to eat fruits and vegetables		
	Provide fruits or vegetables for your child as a snack or part of a meal		
	Eat fruits and vegetables with your child		
	Encourage your child not to drink sugary beverages		
	Talk with your child about the correct portion sizes of the foods to eat		

a ICC=intraclass correlation coefficient.

b TV=television.

c DVD=digital video disc.

d NA=not applicable.

**Table 2:** Hierarchical linear regression of associations between parenting constructs and log transformed sugary beverage consumption among 5- to 8-year-old children participating in Project MOVE/me Muevo (n=539)

		Standardized β				
	*R*^2^ change	Block 1	Block 2	Block 3	Block 4	Block 5
Demographics *R*^2^=0.093						
Child sex		.067	.068	.066	.048	.050
Caregiver age		−.028	−.036	−.037	−.047	−.046
High school education vs middle school		−.155⁎	−.166⁎	−.169⁎⁎⁎	−.194⁎⁎⁎	−.206⁎⁎⁎
Some college education vs middle school		−.195⁎⁎⁎	−.225⁎⁎⁎	−.222⁎⁎⁎	−.221⁎⁎⁎	−.225⁎⁎⁎
College graduate education vs middle school		−.312⁎⁎⁎	−.334⁎⁎⁎	−.331⁎⁎⁎	−.315⁎⁎⁎	−.316⁎⁎⁎
Postgraduate education vs middle school		−.376⁎⁎⁎	−.398⁎⁎⁎	−.389⁎⁎⁎	−.349⁎⁎⁎	−.361⁎⁎⁎
*R*^2^=0.137	0.045					
Parent household rules^a^			−.214⁎⁎⁎	−.156⁎	−.089	−.060
*R*^2^=0.140	0.004					
Parent modeling of rules^b^				−.088	−.069	−.049
Parent-mediated behaviors *R*^2^=0.193	0.063					
Eating dinner together (3.5 times per week vs not)					.013	.006
Eating dinner together (6 times per week vs not)					−.048	−.038
TV on during meals/snacks					.075	.073
Weekly eating away from home at family and friends					.029	.023
Weekly eating away from home at fast-food restaurants					.113⁎	.095⁎
Weekly eating away from home at sit-down restaurants					.055	.058
Screen time					.139⁎	.135⁎
*R*^2^=0.209	0.016					
Parent support						−.142⁎⁎⁎

a Parent rules included the following seven household rules: limited portion sizes at meals, no meals while watching television/digital video discs, no fried snacks (such as potato chips) at home, must eat dinner with family, limited fast food, no sugary beverages, and must finish all food on plate.

b Parent modeling of rules includes the extent to which caregivers followed the same seven household rules set for their children.

⁎ *P*≤0.05.

⁎⁎⁎ *P*≤0.001.
